# Design and Application of an Imprinted Polymer Sensor for the Dual Detection of Antibiotic Contaminants in Aqueous Samples and Food Matrices

**DOI:** 10.1021/acsapm.4c03218

**Published:** 2025-02-19

**Authors:** Oliver D. Jamieson, Jérémy Bell, Alexander Hudson, Joshua Saczek, Victor Pérez-Padilla, Gustavo Kaiya, Katarina Novakovic, Matthew Davies, Emma Foster, Jonas Gruber, Knut Rurack, Marloes Peeters

**Affiliations:** † Department of Chemical Engineering and Analytical Science, School of Engineering, University of Manchester, Manchester M13 9QS, U.K.; ‡ School of Engineering, Newcastle University, Merz Court, Claremont Road, Newcastle Upon Tyne NE1 7RU, U.K.; § Chemical and Optical Sensing Division, Bundesanstalt für Materialforschung und -prüfung (BAM), Richard-Willstätter-Straße 11, 12489 Berlin, Germany; ∥ Departamento de Química Fundamental, Instituto de Química, Universidade de São Paulo, Av. Prof. Lineu Prestes, 748, São Paulo CEP 05508-000, São Paulo, Brazil; ⊥ Departamento de Engenharia Química, Escola Politécnica, Universidade de São Paulo, Avenida Prof. Luciano Gualberto, Trav. 3, 380, São Paulo CEP 05508-000, São Paulo, Brazil; # Newcastle University, Bioimaging Unit, Leech Building, Framlington Place, Newcastle Upon Tyne NE2 4HH, U.K.

**Keywords:** molecularly imprinted polymers, tetracycline, antibiotic monitoring, fluorescence, heat-transfer method, flow cell.

## Abstract

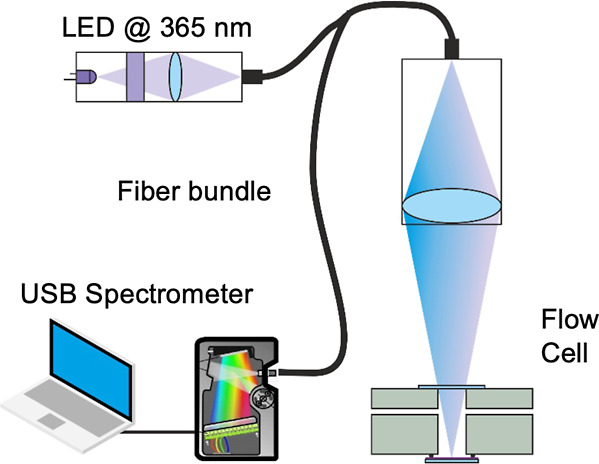

An innovative polymer-based dual detection microfluidic platform has been developed for the accurate and reliable sensing of trace amounts of antibiotic tetracycline in environmental and food samples. This was achieved through the production of a bespoke polymeric material formed via an imprinting technique using a fluorescent dye. Thus, this enables dual detection of tetracycline, both thermally, via analyzing the heat-transfer resistance at the solid–liquid interface, and optically, through the inner filter effect. The combination of these two methods achieved a nanomolar limit of detection for tetracycline while also providing rapid, selective, and cost-effective sensing. Additionally, this method successfully detected tetracycline levels of 0.56 μM in blank egg samples which was significantly lower than the maximum residual level of 400 μg L^–1^ (0.9 μM). Our work shows that this approach can be used for the efficient detection of trace antibiotics in complex environmental and food samples, offering enhanced reliability through the integration of two complementary analysis techniques. This sensor has the potential to identify sources of antimicrobial resistance, which is crucial for targeted efforts to combat this pressing global health challenge.

## 1 Introduction

Tetracyclines (TCs) are a widely used class of broad-spectrum antibiotics, commonly employed in the treatment of bacterial infections in both humans and animals, as well as in agricultural feed additives.^1,2^ However, their extensive use has contributed to the growing problem of antimicrobial resistance (AMR), affecting human and animal health,^3−5^ leading to bioaccumulation within the food chain.^6−8^ This bioaccumulation can have severe health consequences, including direct effects, such as hepatoxicity and renal failure,^9,10^ as well as indirect effects, such as alternations to the gut microbiome.^11,12^ Alarmingly, even foods labeled as “antibiotic-free” have been found to contain trace amounts of antibiotics, including TC, highlighting the critical need for advanced analytical chemistry and sensory solutions to detect these compounds.^13^

Given the persistent nature of TCs and the development of variants,^14,15^ there is a pressing need for innovative detection methods, especially those that can be employed outside conventional laboratory settings.^16−18^ Fluorescence-based sensors are particularly promising due to their inherent sensitivity and ease of miniaturization for field use.^19^ Current rapid and onsite analytical approaches generally fall into two categories: irreversible methods, such as many immunoassay formats that require additional tracers or markers for signal generation,^20,21^ and reversible methods, which use sensor materials that directly embed signaling moieties.^22,23^ Among the latter, molecularly imprinted polymers (MIPs) show great potential for targeting small organic molecules as they can mimic natural receptors while requiring significantly less time for preparation than the generation of antibodies or the organic synthesis of larger artificial supramolecular host molecules.^24−26^

MIPs are porous polymeric materials engineered to bind specific target molecules with high affinity, comparable to antibodies, while offering advantages like low cost, high robustness, and versatility.^27^ For instance, Pizan-Aquino et al. demonstrated the use of magnetic MIPs in an electrochemical TC sensor, achieving significant specificity and validating their approach using high-performance liquid chromatography coupled with UV–vis detection.^28^ However, the complex and time-consuming synthesis of these MIPs limits their commercial viability. In another study, Hudson et al. employed easy-to-produce MIP films for detecting the beta-lactam antibiotic nafcillin using fluorescence and thermal analysis,^29^ though the sensitivity of their fluorescent monomer to environmental factors such as pH poses challenges for real-world applications.

Building on our earlier work, we have focused on optimizing the fluorescent polymer composition,^30,31^ particularly by incorporating 9-vinyl anthracene (9-VA) as the fluorescent monomer, an approach that remains largely unexplored in the context of MIP-based sensors. While Zhang et al. developed a recognition element using 9-VA, they only used it to functionalize carbon nanotubes (CNTs) with vinyl groups and not for signal read-out due to the anthracene moiety π-stack on the CNT surface.^32^ In this work, we introduce a MIP-based platform that utilizes 9-VA for the dual detection of TC in food and environmental samples integrating fluorescence analysis via the inner filter effect (IFE) with thermal detection using the heat-transfer method (HTM).

Fluorescence sensing for small molecules is a vast area with significant previous work. One sensor devised, developed a dual fluorescent MIP probe for the detection carboxylates through the use of MIP recognition shells, incorporating a green-fluorescent BODIPY indicator.^33^ This work afforded a sensor that exhibited a compelling LoD of 0.13 μM and an extensive dynamic range for fexofenadine. The work presented here has taken a different approach through producing thin MIP films on functionalized glass utilizing a commercially available fluorescent monomer (9-VA) resulting in a less complex production for TC which the aforementioned dual fluorescent MIP could not detect due to lack of carboxylate groups and was not able to perform in an aqueous environment.

The IFE is a well-established phenomenon in which the absorption of excitation or fluorescence light by a species in a sample leads to quenching, which can be exploited for detection.^34^ Previous work has shown the simplicity of IFE-based sensors,^35^ including the detection of TC through the use of nitrogen-doped carbon nanodots (CDs) as the fluorescent probes.^36^ However, although IFE-based fluorescence sensing is inexpensive and offers considerable flexibility, its effectiveness can be limited by the availability of suitable analyte–dye pairs and the potential interference from complex sample matrices. Furthermore, the system selectivity is purely based on other species absorption spectra not matching the excitation spectrum of the prepared CD. A recognition element, such as MIPs, can be used to introduce a secondary selectivity element. We combined IFE with HTM, a technique that measures the thermal resistance at the solid–liquid interface. The HTM methodology was initially developed for single-nucleotide polymorphisms in DNA^37^ and has since been adapted for small-molecule detection in complex matrices using MIPs.^38^

This dual-mode sensing system utilizes the strengths of both fluorescence and thermal detection, providing a robust and versatile platform for TC detection. The HTM exploits the specific binding of the target to the MIPs, which creates a measurable insulating effect, while fluorescence analysis detects binding to the MIP through the IFE, where TC absorption of excitation light leads to reduced fluorescence emission. This combined approach of two orthogonal techniques avoids the complexity often associated with other fluorescence-based sensors, such as those relying on Förster resonance energy transfer, and offers a self-validating, dual-analytical technique for the sensitive and selective detection of antibiotics.

**Figure 1:**
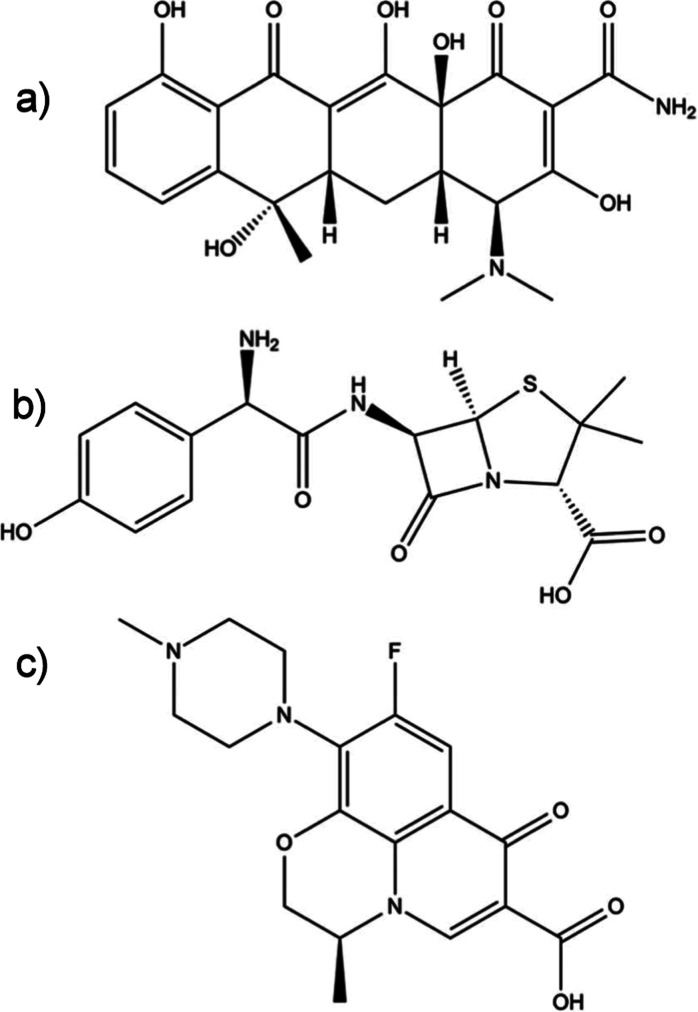
Chemical structures of (a) tetracycline, (b) amoxicillin, and (c) levofloxacin.

In this article, we present the development of our dual detection platform and evaluate its performance in terms of response and selectivity against a range of antibiotics, including amoxicillin and levofloxacin, which represent different classes, such as beta lactams and fluoroquinolones, that contribute to accelerated development of AMR (Figure 1). MIPs are produced using templated polymerization and thus TC was added during MIP production to form binding sites with high affinity and specificity for the selected target. TC was chosen as it is the principal drug from which other analogues were derived, given that contamination to any analogue can lead to the associated environmental or health issues, the pharmacophore is most imperative to detect. Once established for TC, it would require little optimization to extrapolate the sensor to be used on other TC analogues. The work described hereafter provides advancements in the application of MIPs for food safety monitoring. Although the present system is designed for small molecules, with MIPs proven to be able to detect much larger targets, such as yeast (*Saccharomyces cerevisiae*),^39^ this work provides a crucial step for the direct sensing of AMR bacteria in the aqueous and food samples. The resulting sensor system not only meets the demands for rapid, low-cost detection but also provides the reliability and accuracy required for practical, real-world applications.

## 2 Experimental Section

### 2.1 Equipment and Reagents

9-VA and TC were sourced from Alfa Aesar (Heysham, UK) and dimethyl sulfoxide (DMSO) was procured from TCL (Oxford, UK). Levofloxacin (LFX) was acquired from Glentham Life Sciences (Corsham, UK). 3-(Trimethoxysilyl)propyl methacrylate, methacrylic acid (MAA), amoxicillin, trimethylolpropane trimethacrylate (TRIM), azobis(isobutyronitrile) (AIBN), dimethylformamide (DMF), hydrochloric acid (HCL), methanol, chloroform, ammonium hydroxide, hydrogen peroxide, and amoxicillin were all purchased from Thermo Fisher Scientific (Oxford, UK). Phosphate-buffered saline (PBS, 10 mM, pH 7.4) tablets were acquired from Oxoid (Hampshire, UK) and were dissolved in deionized (DI) water (resistivity 18.2 MΩ cm). Chicken eggs were acquired through a local produce vendor. UV polymerization was induced by a Polytec UV LC-5 light source (λ_max_ = 365 nm, Karlsbad, Germany). A FLUOstar Omega spectrometer (BMG Labtech, Aylesbury, UK) was used for initial fluorescence studies. A Nikon Eclipse Ti-E, Nikon (Surbiton, UK) inverted microscope, was subsequently used for fluorescence analysis. A Stuart mini orbital shaker SSM1 (Staffordshire, UK) was used throughout the study. All experiments were carried out at 21.0 ± 1.0 °C unless otherwise stated. During HTM analysis, the temperature was controlled with a VWR INCU-Line Digital Mini Incubator (Lutterworth, UK).

### 2.2 Glass Cleaning and Functionalization Protocol

Glass substrates (10 mm^2^) were sonicated three times at 65 °C for 8 min, first in DI water, followed by methanol, and finally acetone. After drying, the chips were washed separately in the following solutions and sonicated for 15 min each at 65 °C, rinsing with DI water twice between the cleaning solutions. First, with ammonium hydroxide, hydrogen peroxide, and water (1:1:5 volume ratio), followed by hydrochloric acid, hydrogen peroxide, and water (1:1:5 volume ratio). The chips were then thoroughly washed with DI water. After drying, the chips were placed in 4% MAP TMS in toluene (15 mL). The chips in solution were placed onto an orbital shaker and then left for 24 h at 100 rpm. Afterward, the chips were washed with methanol and dried individually with nitrogen.

### 2.3 Synthesis of Molecularly Imprinted Polymers

MIP synthesis was carried out by dissolving TC (0.11 mM) and the functional monomers 9-VA (0.11 mM) and MAA (2.25 mM) in 3 mL of DMSO/DMF (2:1 v/v). Once dissolved, the cross-linker monomer, TRIM (11.15 mM), followed by the initiator, AIBN (0.11 mM), was added. For polymer film production, the reactants were scaled down by a factor of 5, with reactant ratios being kept constant, except for AIBN, which was increased 10-fold (0.05 mM). This solution was subsequently degassed with nitrogen, sealed, and left at 65 °C for 2 h. Once polymerization was complete, the material was extracted from the vial and ground with a pestle and mortar into a fine powder. The target molecule was removed from the resulting powder via iterative extractions with methanol/water (1:1 v/v) under reflux, monitoring the levels of the target in the filtrate spectroscopically. The extraction was then continued until negligible traces of target were present in the filtrate thus indicating extraction was complete, after 1 week due to the highly cross-linked system.

**Figure 2:**
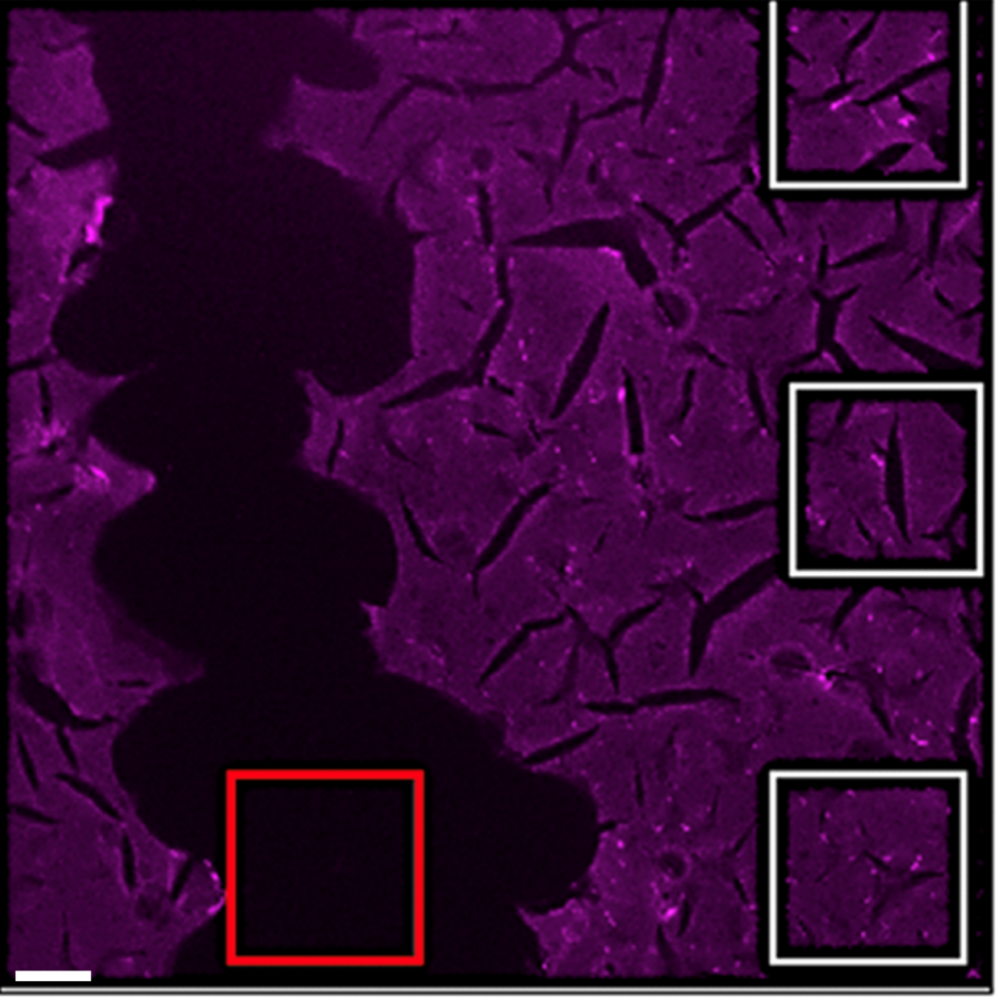
Example region of a polymer-modified glass chip showing both regions covered with polymers (shown by the white boxes) and without (shown by the red box). This is a monochrome fluorescent image, which was colorized for better clarity (scale bar = 200 μm).

Polymer films were synthesized in a similar manner to the aforementioned method; however, lower quantities of the reactants were implemented given the nature of the smaller scale synthesis. This reaction solution was degassed with nitrogen and then sealed completely for later polymerization. The polymerization mixture (8 μL) was added onto the functionalized glass substrates with a microscope slide then placed on top. Chips were polymerized three times, to maximize surface area coverage, for 2 min, 1.5, and 1.25 min, respectively, with a chloroform wash between each polymerization. After the final polymerization, a chloroform wash followed by a methanol wash was carried out on the films. It was found that with methanol having a lower volatility than chloroform, if the final wash was carried out in methanol, the drying process happened at a much slower rate, therefore reducing any cracks or deformation to the films. Extraction of the template molecule was achieved through overnight agitation of the chips in methanol/water (1:1 v/v), and this extraction process was optimized to provide a gentle form of extraction so as not to damage the polymer film. The films can be seen in Figures 2 and S1, showing regions of glass with no polymer present when viewed through the fluorescence microscope (Figure S2).

Non-imprinted polymers (NIPs) served as a reference and were produced according to the same protocol, barring the addition of a target molecule (e.g., TC) in the reaction mixture.

An inverted fluorescence microscope was employed to analyze the coating of the glass substrate by the polymer films. The surface of the chips presented two distinct regions, one polymer-coated section and one uncoated region represented as blank glass as shown in Figure 2, and the blank region established a background control for the analysis. The analysis was set up to run for 10 min with PBS solution flowing into the cell, allowing for an initial reading to be taken at 0 min, with subsequent images captured every 2 min. There was no impact on the initial polymer coating observed due to wetting and applied shear forces.

### 2.4 Batch Rebinding Experiments

TC solutions (in PBS, 5 mL) of varying concentrations were added to MIP powder (10 mg), after which the resulting suspensions were placed in an orbital shaker (100 rpm) for 1 h. The solutions were then filtered, with the filtrate analyzed via spectrophotometry to determine the concentration of the target to the polymer film.
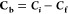
1The concentration was calculated with eq 1, where *C*_*i*_ is the initial concentration (μM), *C*_f_ is the final concentration (μM), and *C*_b_ is the concentration bound to the polymer (μM). *C*_f_ is calculated by division of the gradient parameter from the calibration graph, which was formed by measuring PBS solutions spiked with known concentrations of TC (0, 10, 25, 40, 55, and 70 μM).

### 2.5 3D Printed Flow Cell

**Figure 3:**
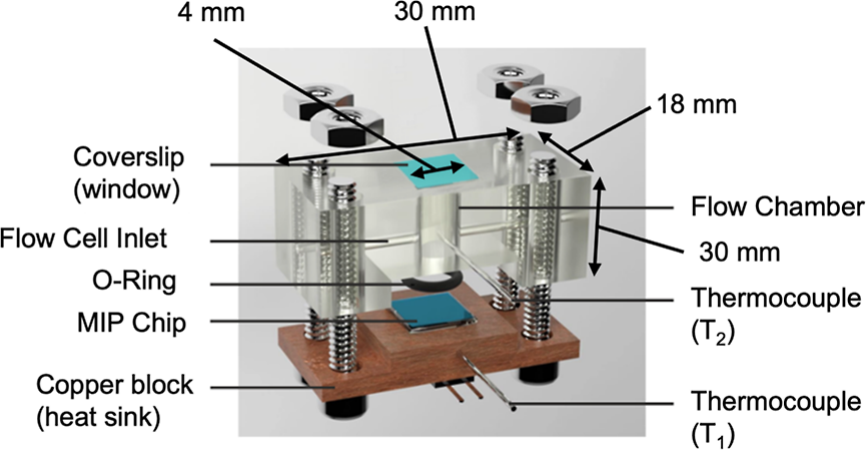
Flow cell design for thermal analysis showing polymer-modified glass chips in between both thermocouples allowing for monitoring of temperature changes at the solid–liquid interface.

For the experiments performed under flow conditions, an in-house resin flow cell was designed and 3D printed (Figure 3). The MIP-modified chips were placed into the flow cell and sealed with an O ring to allow for thermal and fluorescence readouts. Inlet and outlet tubing was attached to either end of the flow cell. PBS solutions were injected through the flow cell to fill the measurement chamber (*D* = 4 mm, *V* = 150 μL) with an internal volume of 110 μL. A blank injection of PBS solution was passed through the cell, after which solutions of increasing concentrations of TC (0, 0.1, 1, 10, 100, and 1000 μM) and other antibiotics (LFX and AMX) were injected. Injections were carried out at a flow rate of 250 μL min^–1^ via an automated NE500 programmable syringe pump (Prosense, Oosterhout, The Netherlands) or a benchtop Chemyx Fusion 100 syringe pump (KR Analytical Limited, Sandbach, UK), with 30 min intervals between each injection for HTM analyses.

### 2.6 HTM Analysis

All thermal analysis experiments were carried out via the HTM coupled with a flow cell with a copper block attached acting as a heat sink. A Proportional-Integral-Derivative (PID) controller was used to regulate the temperature of the copper block (*T*_1_) which was set at 37.00 ± 0.02 °C. These PID parameters were optimized to yield the settings (*P* = 1, *I* = 14, *D* = 0^40^) with the lowest noise on the signal, as the noise critically determines the limit of the detection of the sensor. By subtracting the temperature of the sample (*T*_2_) from the temperature of the copper block (*T*_1_), which was monitored at 1.7 mm above the chip surface, and by dividing this over the power input (*P*) to keep *T*_1_ constant, the thermal resistance (*R*_th_, °C W^–1^) at the solid–liquid interface could be found, as shown in eq 2.
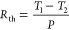
2

### 2.7 Fluorescence Analyses

Initial absorption and emission analyses conducted with the fluorophore were carried out via dissolving 9-VA (0.1 mM) in DMSO/DMF (2:1 v/v) and titrating the solution with increasing aliquots of antibiotics to analyze the template–fluorophore interaction through the use of a spectrophotometer (Specord 210 PLUS, Analytik Jena, Jena) and a fluorometer (Fluoromax 4, HORIBA, Darmstadt).

**Figure 4:**
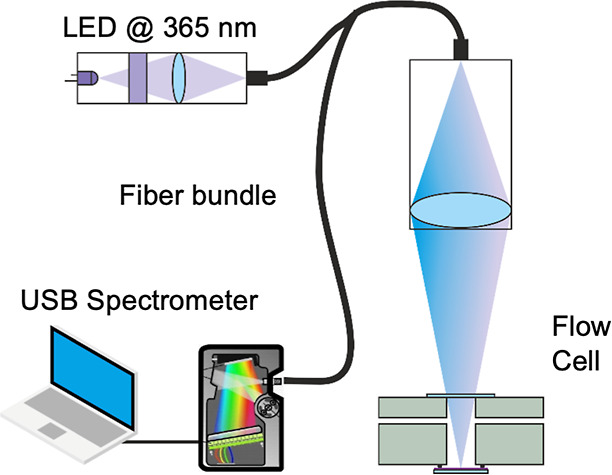
Scheme of the miniaturized fluorescence readout system adapted to the flow cell using a LED excitation source, an optical fiber bundle, and a USB spectrometer.

The fluorescence signal in the cell was measured with a custom-built optical excitation/emission setup (Figure 4) using a 6 + 1 multimode fiber bundle (Ø_cores_ = 200 μm, FRP-200-0.22-1, B&W Tek, Filderstadt).

A LED with emission at 365 nm (XSL-365-5E, Roithner Lasertechnik, Vienna) was used for excitation of the polymer chip inside the flow cell. Its main beam was focused into the illumination fiber with a convex lens (f = 15 mm, LA1540, Thorlabs, Bergkirchen) through a 365 ± 10 nm bandpass filter (#65-069, Edmund Optics, Mainz). The excitation output from the fiber was focused through the flow cell chamber onto the chip via a convex lens (f = 35 mm, LA1805, Thorlabs). The same lens was used to collect back the emission from the chip into the six pick-up fibers. The bundle outlet was connected to a USB2000+, ILX511B spectrometer (OceanOptics, Ostfildern) and signal acquisition was made on a PC computer via the SpectraSuite software.

### 2.8 HTM Analysis of Egg Samples Spiked with TC

Thermal analysis via the HTM was then carried out on a TC-spiked egg solution in a manner similar to that previously described, with adaptation of the cell from a continuous to a batch system to avoid issues with handling of viscous sample matrixes, such as eggs (Figure S3). Eggs were homogenized (vitellus and albumen) which were then diluted in PBS in a 1:3 ratio. This egg suspension acted as a blank reference for the thermal analysis and was stabilized for 15 min, followed by the introduction of 150 μL of the same egg solution but spiked with TC (0.56 and 5.6 μM) to the MIP-modified glass films. The thermal resistance was monitored over time to determine if the developed MIP-based sensor was capable of monitoring relevant levels of TC in complex food samples.

## 3 Results and Discussion

### 3.1 Analysis of MIP

#### 3.1.1 Optical Batch Rebinding Experiments

Specificity studies were carried out on an MIP prepared with TC as the template (MIP T) and a reference NIP to gain insight into binding characteristics of the polymer (Figure S4). At a concentration of 40 mM, there was a significant difference between the binding of the MIP T and NIP, which further increased until the highest concentration of 70 mM. This confirmed the specificity toward TC of the chosen polymer composition, which was further interrogated with fluorescent and thermal analysis.

#### 3.1.2 Fluorescence Analysis

**Figure 5:**
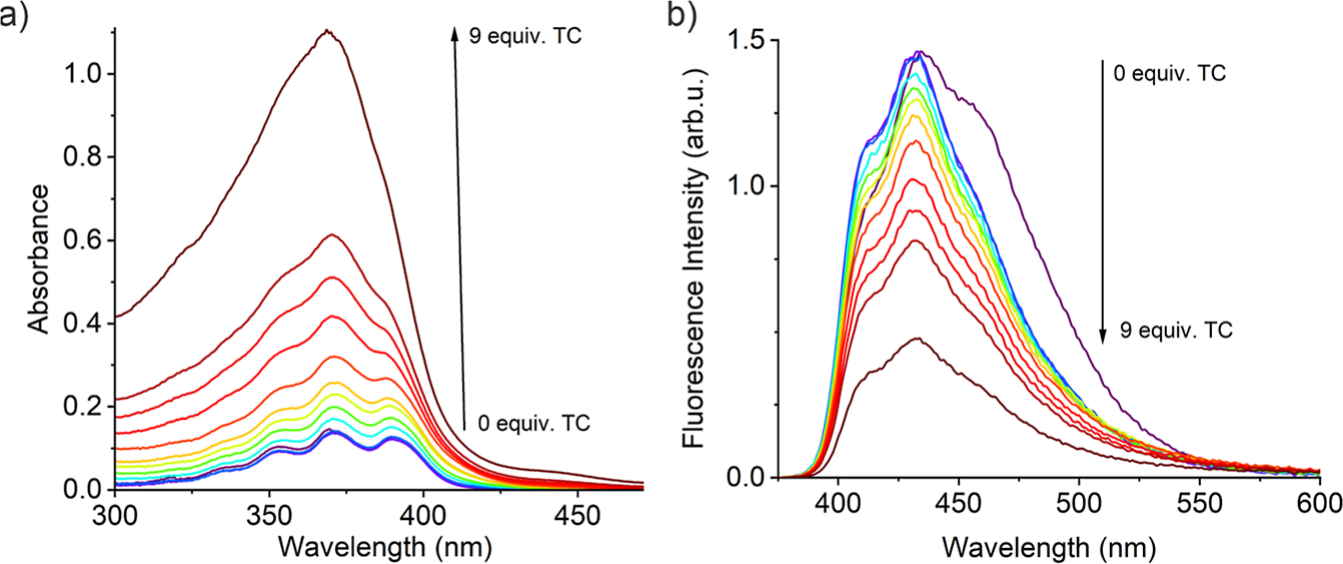
9-VA absorption (a) and emission (b) spectra in DMSO/DMF (2:1 v/v) upon addition of increasing equivalents of TC (*c*_9-VA_ = 10 μM, λ_exc_ = 360 nm).

Prior to the analysis of the polymer, photophysical studies were carried out on the fluorescent monomer (9-VA) in the presence of various antibiotics. Comparing the emission of 9-VA, TC, LFX, and AMX and combinations thereof allowed us to establish a mechanistic understanding of the observed optical phenomena constituting the sensing response. The absorption and emission spectra of the single species (Figure S5) as well as 9-VA in the presence of increasing aliquots of TC were recorded (Figure 5).

The absorption spectrum of 9-VA showed a typical anthracene-like band with a global maximum at 370 nm. This band overlaps almost completely or partly with the absorption band of TC and LFX but not with the absorption band of AMX, presenting an absorption maximum deeper in the UV spectrum at 275 nm. The emission band of all studied species was observed in the blue/green range with an emission maximum for 9-VA at 435 nm. According to those observations, especially the overlap of the absorption of some of the antibiotics with that of 9-VA, we expect that increasing concentrations of TC or LFX (to a lower extent) in solution would attenuate the excitation beam. Therefore, this leads to a primary IFE and a lower detection of anthracene fluorescence emission. Indeed, the addition of TC to a solution of 9-VA caused a continuous decrease in the fluorescence intensity. This net quenching effect is due to the fluorescence quantum yield of TC being much lower than that of the anthracene derivative; even for high TC concentrations, no band at 550 nm was observed under those conditions. This suggested that TC can be quantified thanks to the attenuation of the intense fluorescence of 9-VA via an IFE also when 9-VA was embedded in a MIP.

9-VA-based MIPs were thus attached to functionalized glass chips for fluorescence studies inside the 3D-printed cell with a custom-built fluorescence reader. The MIP was directly polymerized onto the surface of the glass. Three layers of polymer were polymerized onto each chip to gain a satisfactory coverage while also increasing the stability and durability of the polymer.

Next, a customized modular setup was utilized to enable better applicability and specificity of the sensor (Figure 4). This setup allowed for excitation of the polymer film within the flow cell from the opposite side of the copper heat sink, allowing for a continuous flow measurement cell applicable to both methods which allows for detection optimization, e.g., faster equilibration times.^41^ The emission signal was collected with a fiber bundle and a USB spectrometer allowed for real-time spectral acquisition to isolate the fluctuating signal of the anthracene monomer from residual excitation light, daylight interferences, or even emission from the analytes. A non-negligible reduction in fluorescence intensity of a polymer film was witnessed when placed inside the flow cell due to partial filtering of the UV excitation by the glass top window and some cutting effects from the flow-cell edges. Nonetheless, the fluorescence signal was intense enough to allow for analysis as shown in Figure S6. Initially, the cell was filled with PBS solution, which was flowed for 10 min before recording the chip’s background signal (*S*_0_) that allows for differences between each manufactured chip. Division of the analytical signal, *S*, by *S*_0_ further provides a chip-independent reading for better comparison of results obtained with different chips or on different days via *S*/*S*_0_. For each data point, the signal was acquired for 5 min and three repetitions were made to calculate the LoD using the total relative error^42^ and the formula described by Armbruster and Pry.^43^

**Figure 6:**
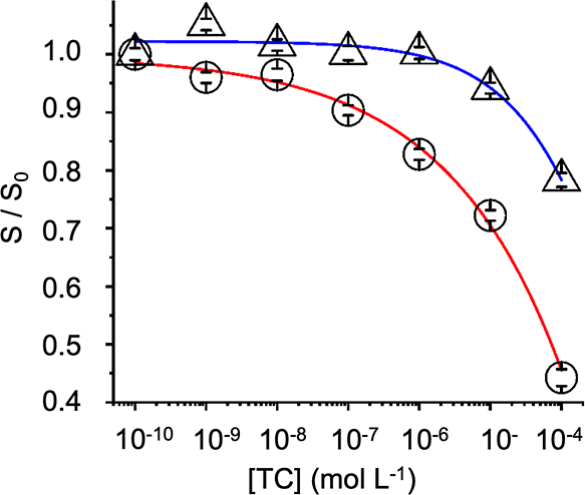
Measured MIP T fluorescence quenching upon addition of TC (circles) along with PBS-mediated recovery of the signal (triangles). Red and blue lines show the good correlation of logistic fits of the calibration and the recovery curve.

When the concentration of TC in the solutions passing through the flow cell was increased, in accordance with the steady-state analyses using 9-VA, an attenuation of the emission of MIP T due to the IFE was observed (Figure 6). Transversely, when the system was flushed with a blank PBS solution, the signal returned to its initial intensity up to *c*_TC_ ∼ 1 μM, indicating that the analyte was not excessively accumulated in the flow cell due to nonspecific interactions.

The calibration curve obtained for the MIP T chips allowed for the detection of TC down to 1.7 nM in aqueous solutions with an efficient signal quenching of up to 56% for a concentration of 100 μM TC. Similar experiments were carried out with LFX and AMX solutions (Figure S7), with the IFE also occurring for LFX but not for AMX, which does not absorb at the excitation wavelength. As such, our optical channel presents a distinct advantage in terms of sensitivity, with detection possible within the nanomolar range, while also producing an almost instantaneous response (<10 s) and thereafter only requiring a short analysis time (5 min).

#### 3.1.3 HTM Analysis

**Figure 7:**
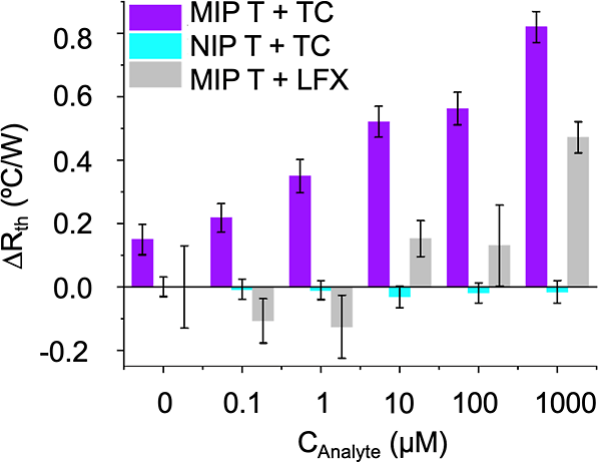
Selectivity and specificity study of the thermal resistance of MIP T and NIP upon injections of increasing TC and LFX concentrations.

Thermal analysis was conducted on both MIP T and its reference polymer to assess the impact that TC solutions of varying concentrations (0, 0.1, 1, 10, 100, and 1000 μM) had on the heat-transfer characteristics of the system. Initially, injections of PBS solution were used as the blank stabilization for the analysis. This allowed for a thermal resistance baseline of the polymer layer on the functionalized glass chip to be established (Figure 7). Once stabilization had occurred, solutions with increasing TC concentrations were injected into the flow chamber. TC solutions were given 20 min for stabilization before the next injection, for the raw data detailing the injections (Figure S8). Following each injection of TC solution into the presence of the reference polymer NIP, no significant change of thermal resistance was observed, even at the highest concentration (1 mM, Figure 7). Taking into consideration that the NIP had the same functional monomer present during polymerization which would be capable of forming interactions between the polymer and TC, the lack of TC-specific cavities proved vital in order to promote specific binding. The HTM analysis exhibited a total relative error of approximately 1%, affording an LoD of 0.54 μM.^43^ This demonstrated that through thermal analysis, the nonspecific binding of TC to the surface of the polymer had no impact on the heat transfer at the solid–liquid interface. Similarly, MIP T was subjected to increasing concentrations of LFX (Figure S9). When the imprinted polymer (MIP T) was observed over the same concentration range of both analytes, there was a significantly higher increase in resistance on each injection of higher concentration TC than LFX, resulting in a discrimination factor of 2.3 (at 1 μM TC and LFX) calculated as described in previous work.^44^ The discrimination factor was calculated at 1 μM of both compounds as previous findings have shown residual aqueous TC concentrations equating to 5 μM in the environment,^45^ so the chosen concentration gives relevance to likely concentrations found in the environment. The highest concentration of TC (1 mM) resulted in a change of 0.82 °C W^–1^ compared to a change of 0.47 °C W^–1^ for LFX. After a final PBS wash, there was no significant change in the *R*_th_, confirming that the change in *R*_th_ was due to the specific binding of the TC to the imprint sites and not nonspecific surface binding which would have been undone during the blank wash. LFX had an effect on the *R*_th_ due to the hydrogen-bond receptors and donors in its structure, thus enabling one to make use of functional binding on the polymer film surface; however, as the structure is different, the level of binding is limited, and therefore a weaker response is seen than when compared to the TC binding. This affirms a significant level of selectivity exhibited by the polymer when subjected to thermal analysis.

We anticipate that the combination of the fluorescence-based technique with the HTM would provide a powerful approach to molecular detection, exploiting the strengths of both methods. The fluorescence technique offers high sensitivity and rapid response, detecting trace amounts of analytes, while the HTM enhances specificity and precision by measuring changes in thermal resistance, ensuring accurate detection even in complex samples. In the future, these two methods could complement each other, with fluorescence offering rapid screening and HTM providing confirmation of specific binding and minimizing nonspecific interactions. To combine both techniques into a singular simultaneous device, the HTM device would compose a change to a much smaller pico-data logger and a portable fluorescence spectrometer. Given that there are large areas of the polymer film surface available during the thermal analysis, fluorescence analysis can be carried out concurrently. This combination and miniaturization opens up significant possibilities for the final device to be portable and simultaneous, affording a multifaceted approach to infield testing. Previous work has already shown that the operational temperature of the HTM analysis can be altered without significant drawbacks, which showcases the device use in varied climates of which an infield testing system would require.^46^

#### 3.1.4 Thermal Analysis of Antibiotics in Egg Samples

This proof-of-concept analysis demonstrated the system validity for real food samples. Antibiotics are a common additive to animal feed that can lead to their bioaccumulation in livestock. Therefore, to increase the commercial applicability of the presented sensor, its ability to test in livestock-related matrices was necessary. The presence of antibiotics within poultry, especially eggs, has been commented on since the 1950s, with recent findings suggesting that all major groups of antibiotic residues, including beta-lactams (amoxicillin) and TCs, have been reported within egg matrices.^47^ Changes in thermal resistance following the introduction of injection 2 and 3, 0.56, and 5.6 μM TC, respectively, to a 1:3 egg/PBS solution (injection 1) and the subsequent comparison against a blank egg/PBS solution (injection 4) can be observed (Figure S10 and Table 1). Error bars were calculated via taking the standard deviation of three independent measurements that were conducted with fresh egg samples and freshly prepared chips. A change in the cell design to a batch system (an addition cell) was required, as the viscous nature of the sample matrix was not compatible with the flow cell setup.

**Table 1:** Change in Thermal Resistance on Injection of TC in Egg Samples

injection	concentration of TC (μM)	Δ*R*_th_ (°C W^–1^)
1	0	0 ± 0.05
2	0.56	0.28 ± 0.05
3	5.6	0.53 ± 0.05
4	0 (wash)	0.52 ± 0.05

MRL TC concentrations in eggs are 0.9 μM, which is much higher than what was detected in this body of work, thereby showing that the sensor engineered can detect trace contamination in food samples.^48^ As outlined in Table 1, the complex egg solution does instigate an increase in thermal resistance; however, it also dampens the response of TC binding to the imprinted cavities in the polymer. Dampening of response due to the matrix effect is not an uncommon occurrence and has been reported upon previously.^37,49^ There is a significant difference between the egg solution and both TC egg/PBS solutions, 52% increase of *R*_th_ when comparing 5.6 μM and blank egg solution, suggesting that the system could be optimized and developed for commercial use for antibiotic contamination detection in eggs, such as a higher operation temperature to reduce viscosity, and this can be further seen in Figure S10. The lack of change in *R*_th_ after the final PBS wash further confirmed that there was significant, specific, and lasting binding of the TC in the egg matrices to the MIP T sites. While there are other factors that can be problematic for sensor measurements, such as pH, salinity, and total suspended solids, the sensors’ ability to detect in a highly viscous sample medium, such as eggs, is a significant attribute even more so when all three parts of the egg were present (the albumen, chalaza, and the yolk) reducing the sample processing required even with a significantly complex medium. These results combined with previous work sensing COVID-19 in clinical samples^50^ demonstrate the ability of MIPs to operate efficiently in a host of different sample media.

## 4 Conclusions

Our study demonstrates the development and application of a low-cost and highly specific imprinted polymer sensor capable of dual detection of antibiotic contaminants in environmental and food matrices. By integrating thermal and optical analysis within a custom-designed measurement cell, we streamlined the hardware requirements, making the system adaptable for portable, in-field sensing, even in remote and resource-limited areas. The bespoke measurement cell allows for both analysis techniques to operate in the same experimental setup, which combines the advantages of fast fluorescent analysis with the high sensitivity of the thermal technique. Thus, the complementary nature allows for future work to develop a simultaneous dual detection system that will significantly enhance the reliability and functionality of the sensor platform. The thermal analysis showed a significant increase in thermal resistance (0.82 °C W^–1^) upon the introduction of 1 mM of TC, while fluorescence analysis enabled the rapid detection of TC at low concentrations, with a 56% attenuation of the MIP’s emission intensity at 100 μM. The system accurately detected TC concentrations in spiked egg samples below the maximum residual level of 0.9 μM, highlighting its effectiveness for real-world applications.

The integration of dual orthogonal analytical techniques not only enhances the reliability and sensitivity of the sensor but also opens new avenues for commercially viable sensing platforms. Future work will focus on optimizing the compatibility between the thermal and optical methods, particularly through enhancing microfluidic platform performance, for smaller volume and more efficient solid–liquid extraction by the sensor membrane and incorporating an optical channel into the heat sink to make use of the direct fluorescence response from the MIP instead of IFE. Since the HTM has previously been shown to detect larger macromolecules, including microorganisms like yeast and bacteria, it holds the potential for simultaneous, real-time monitoring of both antibiotics and bacteria, a capability not achievable with conventional methods. This advancement could further broaden the sensor’s applicability, paving the way for comprehensive monitoring solutions in the fight against AMR.

## Supporting Information Available

Additional figures and diagrams to offer aid in understanding the experimental setup, additional cell adaptions, and foundational and raw data (PDF)
