# Supplementary material for: Design and Application of an Imprinted Polymer Sensor for the Dual Detection of Antibiotic Contaminants in Aqueous Samples and Food Matrices
Source: ACS Appl Polym Mater. 2025 Feb 19;7(4):2265–73. doi: 10.1021/acsapm.4c03218 (PMC11877416; doi:10.1021/acsapm.4c03218)
Supplement: Supplementary file 1 — ap4c03218_si_001.pdf [file ap4c03218_si_001.pdf]

Supporting Information

Design and Application of an Imprinted Polymer Sensor for the Dual Detection of Antibiotic Contaminants in Aqueous Samples and Food Matrices

Oliver D. Jamieson,[1,2] Jérémy Bell,[3] Alexander Hudson,[2] Joshua Saczek,[1,2] Victor Pérez-Padilla,[3] Gustavo Kaiya,[4,5] Katarina Novakovic,[2] Matthew Davies,[2] Emma Foster,[5] Jonas Gruber,[4,5] Knut Rurack,[3] Marloes Peeters[1,2*]

1) Department of Chemical Engineering and analytical science, School of Engineering, University of Manchester, Manchester, M20 4 BX, United Kingdom

2) Newcastle University, School of Engineering, Merz Court, Claremont Road, Newcastle Upon Tyne, NE1 7RU, United Kingdom

3) Chemical and Optical Sensing Division, Bundesanstalt für Materialforschung und -prüfung (BAM), Richard-Willstätter-Straße 11, 12489 Berlin, Germany

4) Departamento de Química Fundamental, Instituto de Química, Universidade de São Paulo, Av. Prof. Lineu Prestes, 748, CEP 05508-000 São Paulo, SP, Brazil

5) Departamento de Engenharia Química, Escola Politécnica, Universidade de São Paulo, Avenida Prof. Luciano Gualberto, trav. 3, 380, CEP 05508-900 São Paulo, SP, Brazil

6) Newcastle University, Bioimaging Unit, Leech Building, Framlington Place, Newcastle Upon Tyne, NE2 4HH, United Kingdom.

Corresponding author: Marloes Peeters, marloes.peeters@manchester.ac.uk

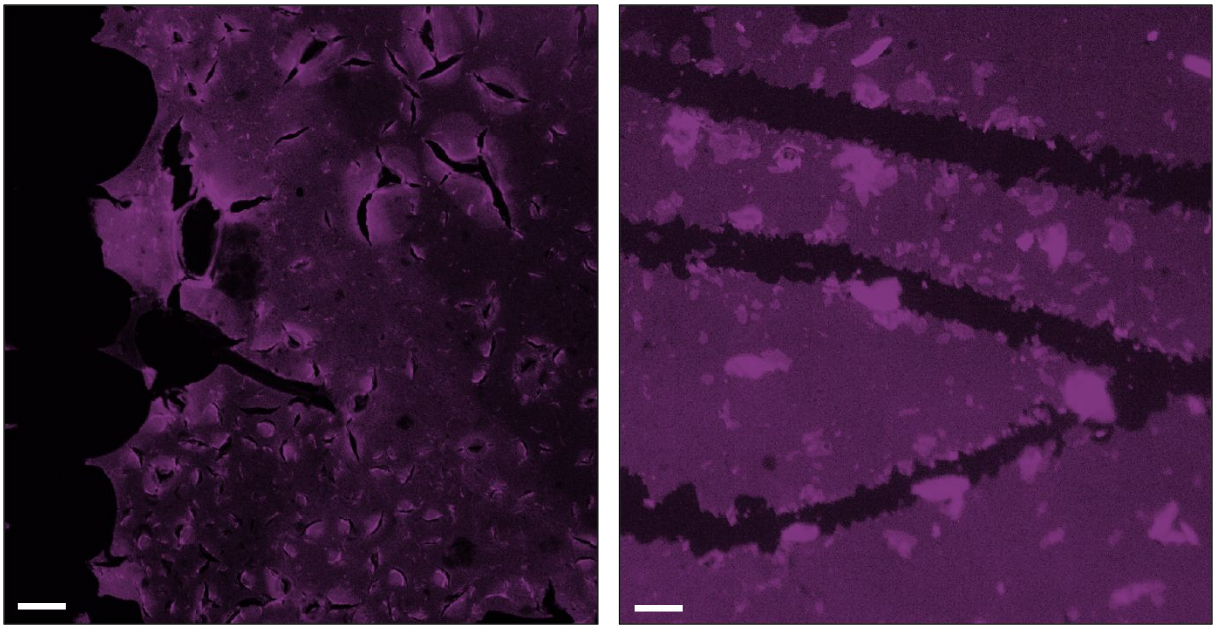

S 1- Capture of MIP films polymerised onto functionalised glass. Scores have been cut through the polymer to act as reference points. These are monochrome fluorescent images, that were colorized for better clarity. (scale bar = 400 μm)

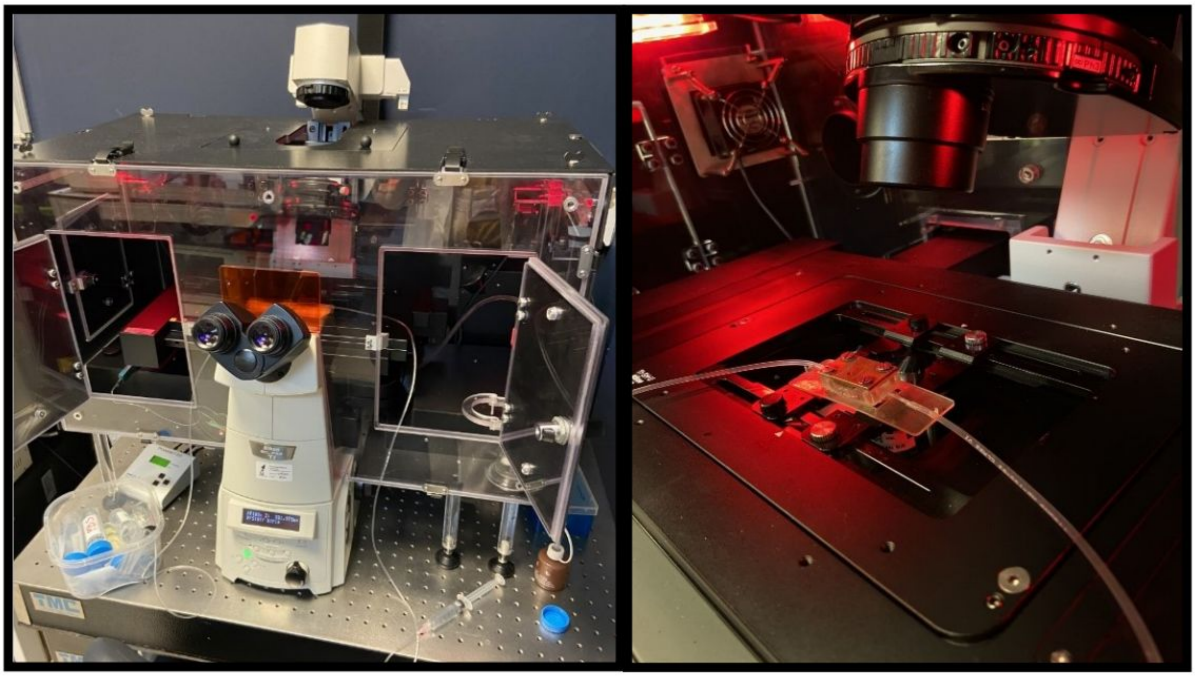

S 2- Analysis set up with polymer chip encapsulated in a printed cell inside a blackout box fitted with an inverted fluorescent microscope.

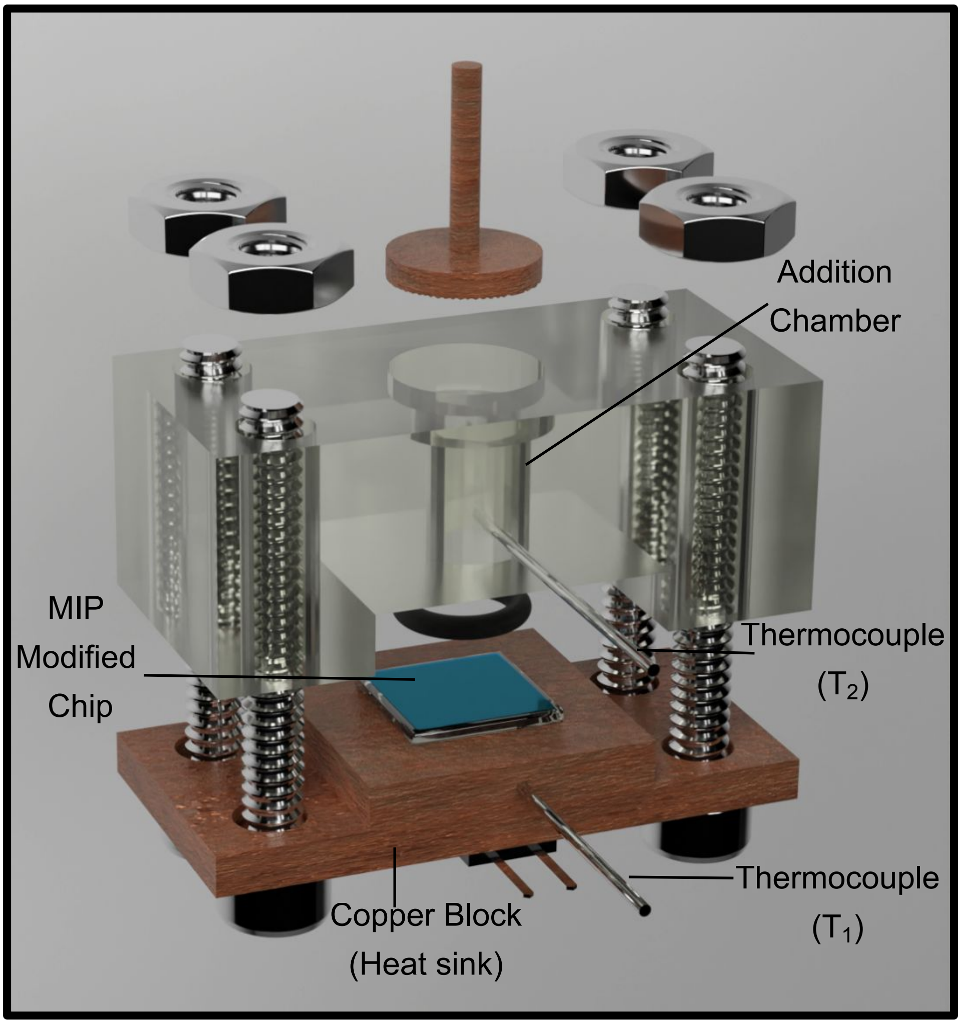

S 3- Addition cell design for the analysis of egg samples.

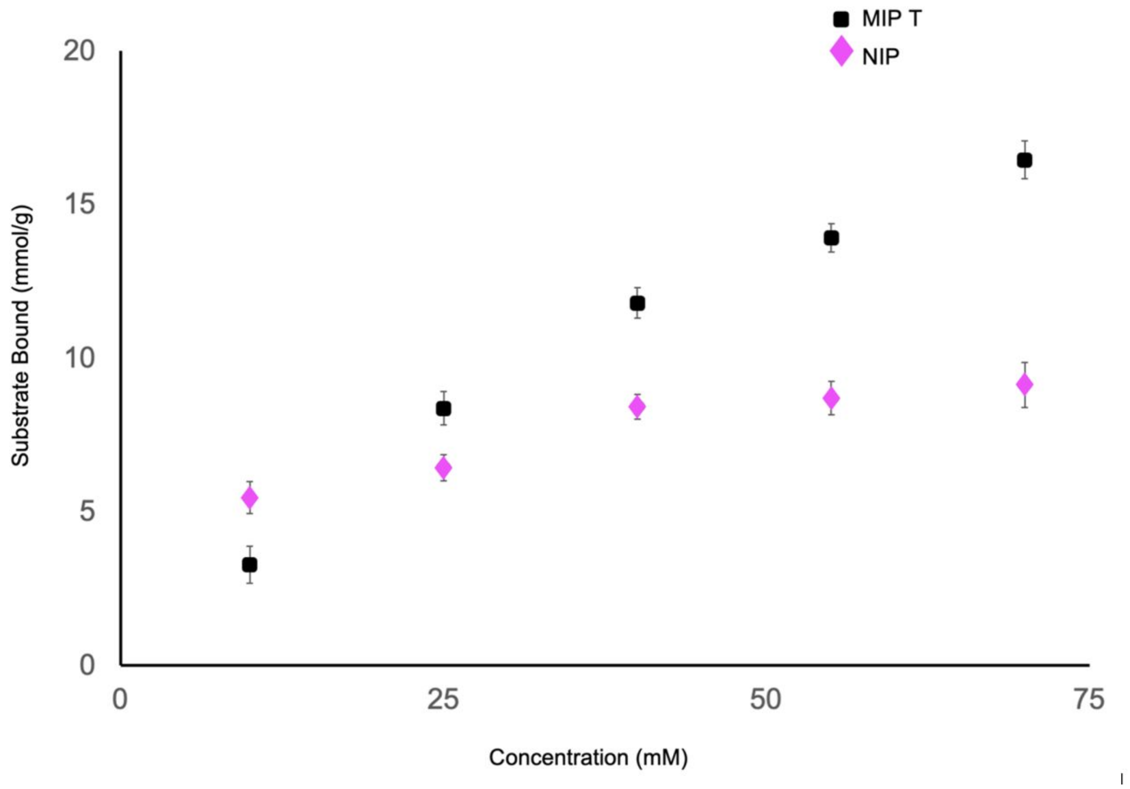

S 4- the specificity study of MIP T and NIP (using 10, 25, 40, 55 and 70 mM TC solutions)

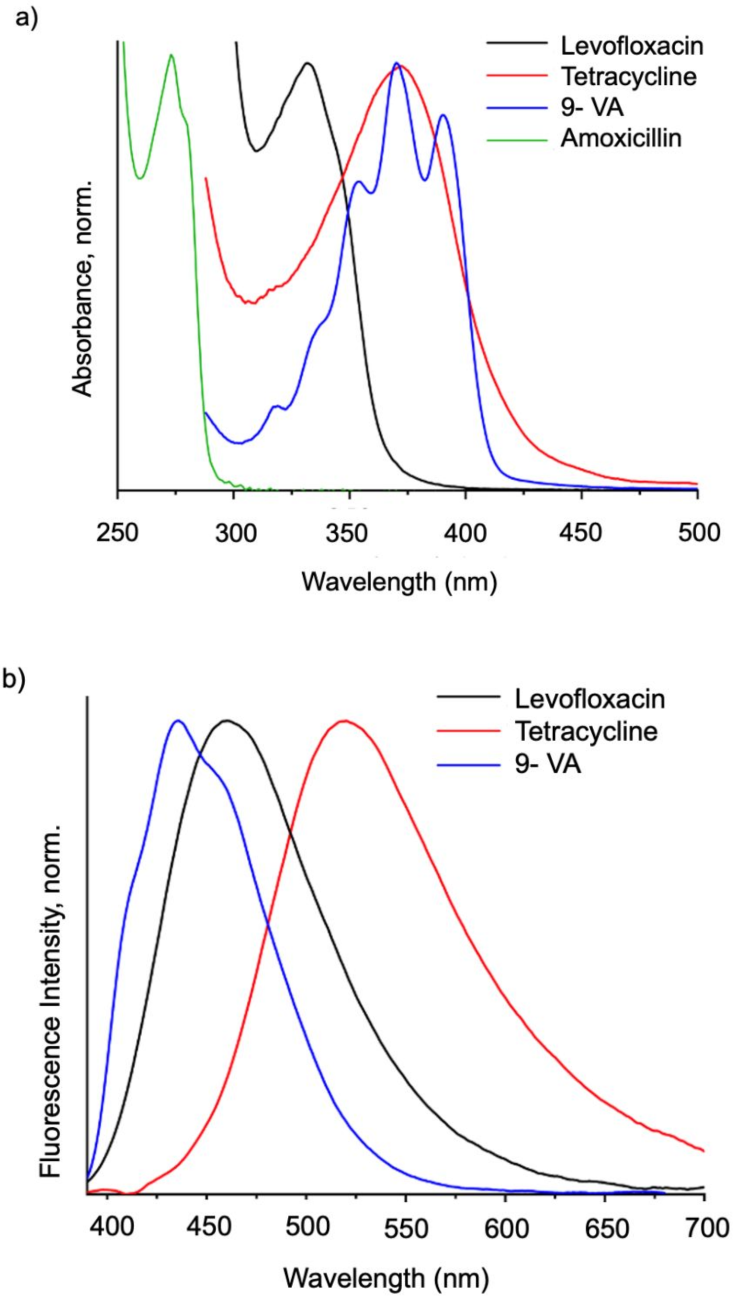

S 5- a) Normalized absorption spectra of 9-VA, tetracycline, levofloxacin and amoxicillin. b) Normalised emission spectra of 9-VA, tetracycline, levofloxacin (λ_{exc} = 360 nm). Amoxicillin does not show any emission signal.

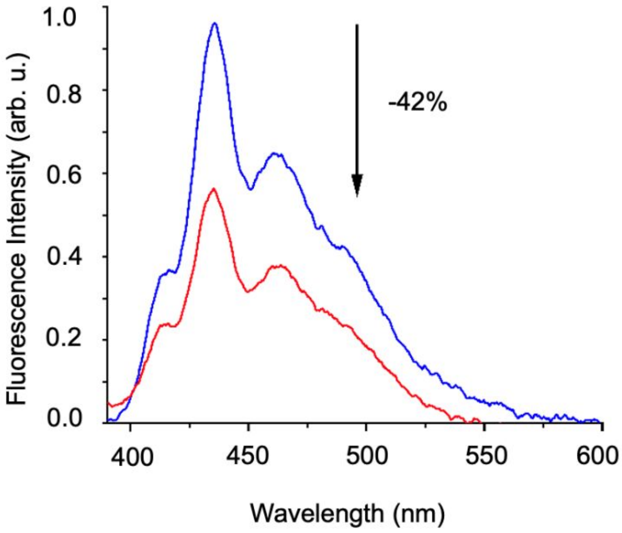

S 6- Quantification of the amount of fluorescent intensity lost through the microscope slide.

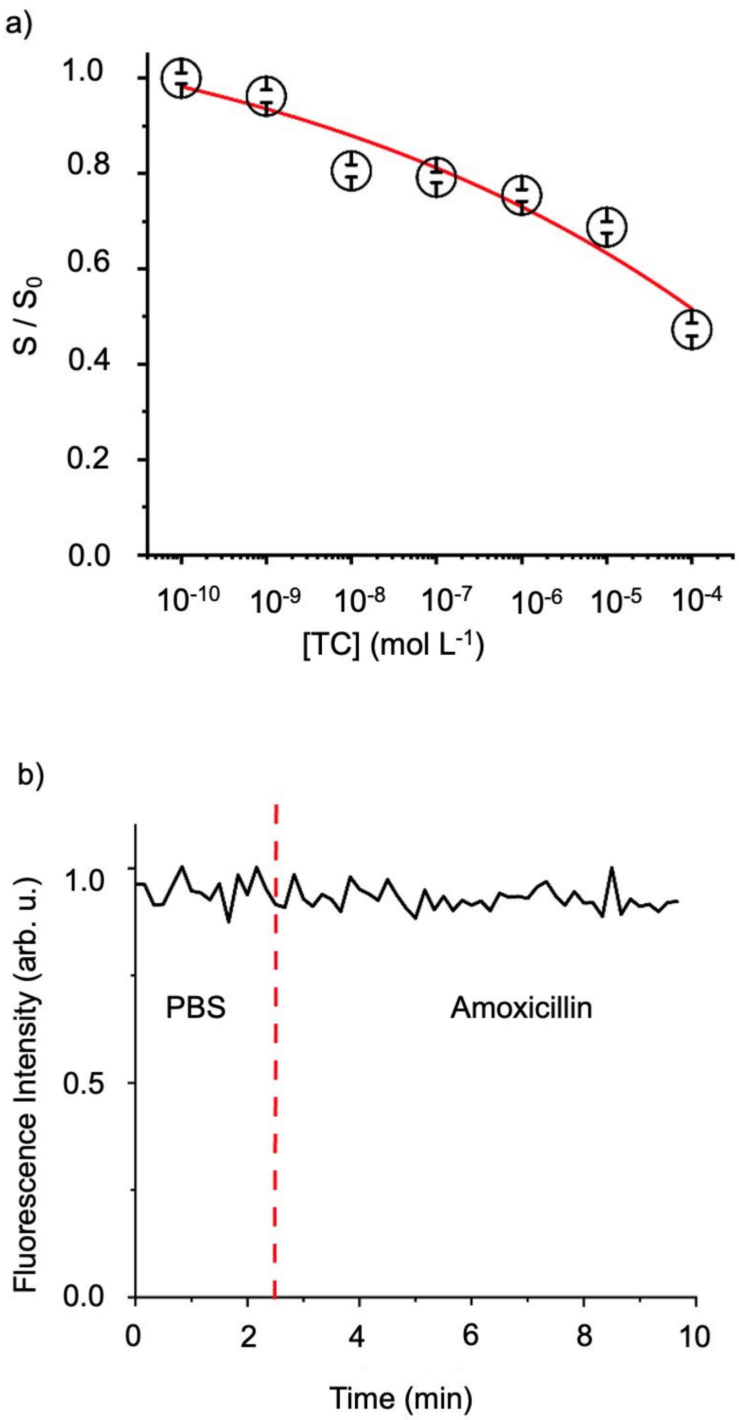

S 7- a) MIP T fluorescence quenching on levofloxacin additions (Logistic fit) and b) MIP T fluorescence stability upon 100 µM amoxicillin addition (dashed red line).

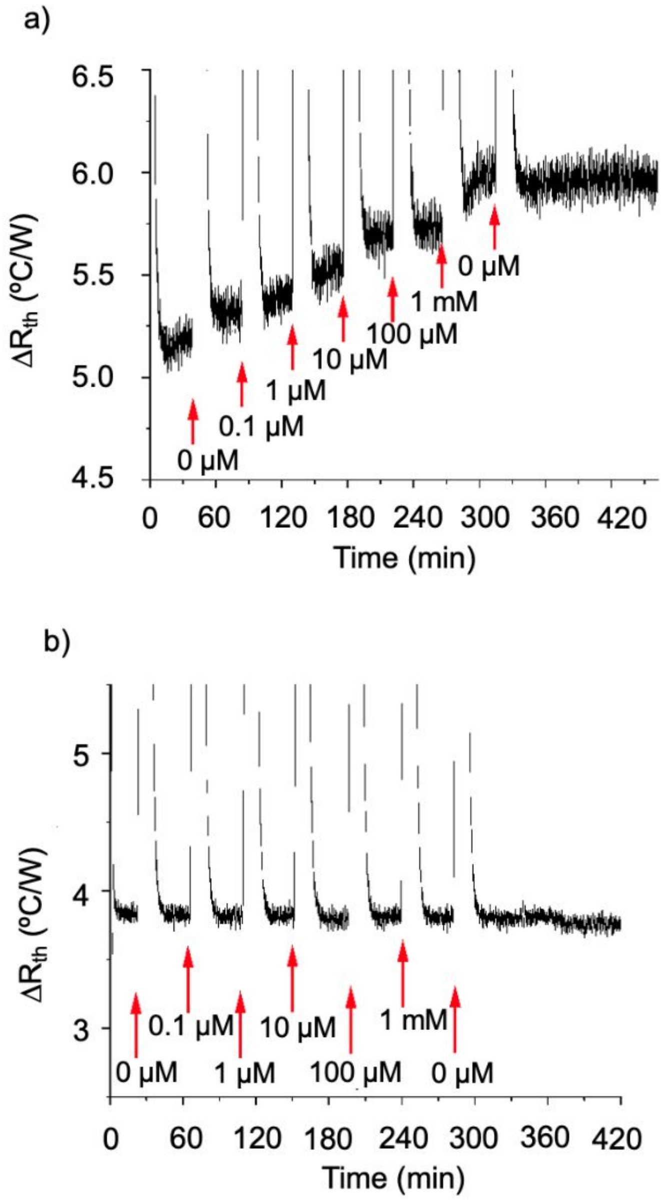

S 8- Raw R_{th} data of a) MIP T and b) NIP when subjected to increasing concentrations of TC.

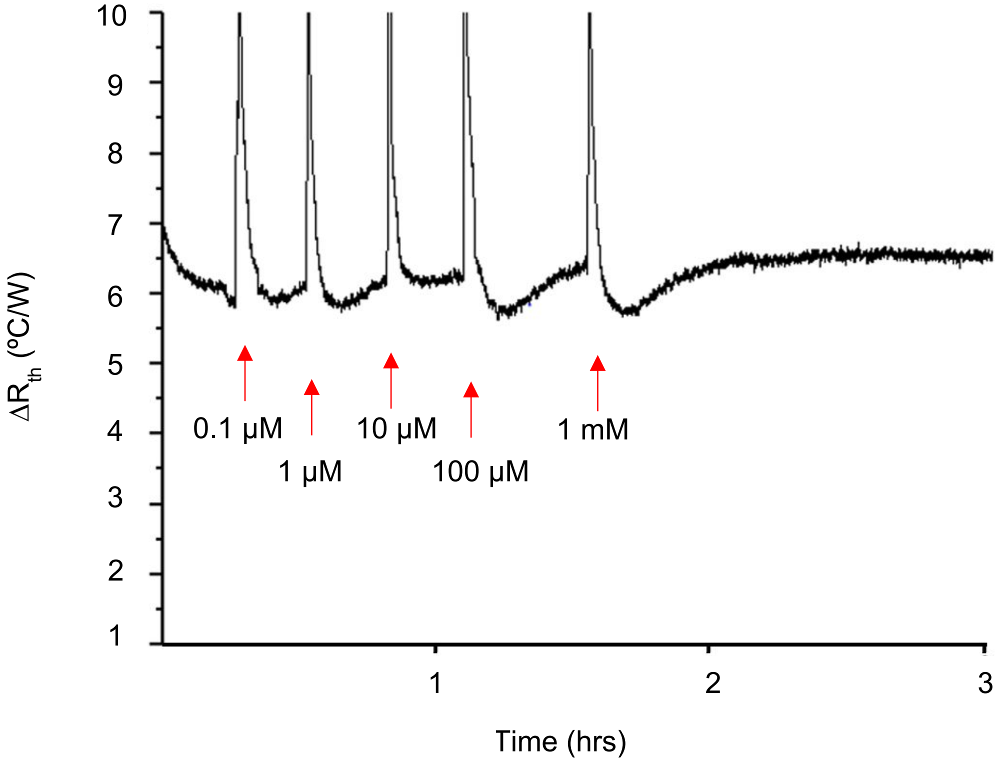

S 9- Thermal resistance of MIP T upon injections of solutions with equivalent increasing levofloxacin concentrations.

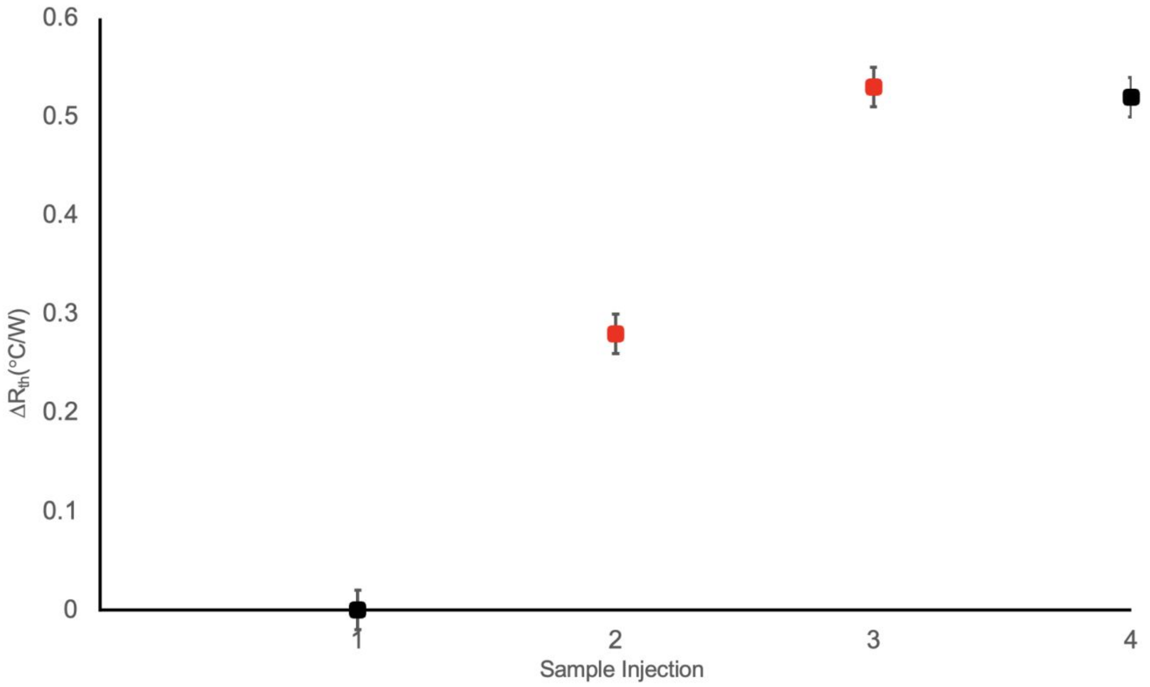

S 10- Change in thermal resistance on injection of (1) PBS, (2) 0.56 µM TC, (3) 5.6 µM TC and (4) PBS in egg solution.
